# The Impact of PM2.5 on the Growth Curves of Children's Obesity Indexes: A Prospective Cohort Study

**DOI:** 10.3389/fpubh.2022.843622

**Published:** 2022-03-22

**Authors:** Jishuang Tong, Yanling Ren, Fangchao Liu, Fengchao Liang, Xian Tang, Daochao Huang, Xizhou An, Xiaohua Liang

**Affiliations:** ^1^Department of Clinical Epidemiology and Biostatistics, Ministry of Education Key Laboratory of Child Development and Disorders, National Clinical Research Center for Child Health and Disorders, Chongqing Key Laboratory of Pediatrics, Children's Hospital of Chongqing Medical University, Chongqing, China; ^2^Department of Epidemiology, Fuwai Hospital, National Center for Cardiovascular Diseases, Chinese Academy of Medical Sciences and Peking Union Medical College, Beijing, China; ^3^School of Public Health and Emergency Management, Southern University of Science and Technology, Shenzhen, China

**Keywords:** air pollution, obesity, obesity indicators, growth curve, children and adolescent

## Abstract

**Aims:**

To explore the effect of long-term exposure to particulate matter with an aerodynamic diameter of 2.5 μm or less (PM2.5) on childhood obesity based on a cohort study in Chongqing.

**Methods:**

A total of 4,284 children aged 6–8 years at baseline were enrolled from the Chongqing Children Health Cohort in 2014–2015 and were followed up in 2019. A stratified cluster sampling was applied to select the participants. A Mixed-effects linear regression model was used to examine the effect of long-term exposure to PM2.5 on the growth curve of obesity indicators [including body mass index (BMI), BMI Z-score (BMIz), and waist-to-height ratio (WHtR)]. A mixed-effects logistic regression model was used to study the dose relationship between PM2.5 exposure and the risk of obesity indicators.

**Results:**

A higher level of accumulating exposure to PM2.5 was associated with an increased childhood obesity index, and the effect was the most significant for WHtR than BMI and BMIz. This effect was more pronounced in boys than in girls except for WHtR, and it was the most significant under the PM2.5 exposure period from pregnancy to 6 years old. Compared the annual average PM2.5 exposure level of <60 μg/m^3^, the WHtR and BMI were increased by 0.019 [(95% CIs): 0.014, 0.024] and 0.326 [(95% CIs): 0.037, 0.616] Kg/m^2^ for participants living with the PM2.5 exposure level of 70–75 μg/m^3^, respectively. For every 5 μg/m^3^ increase in PM2.5 levels (from pregnancy to 6 years old), the risk of central obesity was increased by 1.26 {odds ratio [OR] (95% CIs): 1.26 (1.16, 1.37), *p* < 0.001} times.

**Conclusions:**

This study confirmed a dose-response relationship between PM2.5 exposure and childhood obesity, especially central obesity, suggesting that controlling ambient air pollution can prevent the occurrence of obesity in children and adolescents.

## Introduction

The global prevalence of childhood obesity has increased dramatically in the past decades, however, the majority of adulthood obesity was originated from childhood (1). Children and adolescents with obesity will induce many adverse health events in adulthood, such as type 2 diabetes, hypertension, and cardiovascular disease (2). In 2014, our previous study found that the prevalence of children with overweight and obesity in Chongqing was 13.36 and 8.6%, respectively (3), which will lead to an enormous disease burden. In recent years, air pollution in China remains severe, far exceeding the air quality standards issued by WHO (4). The population-weighted annual average exposure to particulate matter with an aerodynamic diameter of 2.5 μm or less (PM2.5) in China in 2017 was 52.7 μg/m^3^. In total, 81% of the population lives in areas exceeding the WHO interim target (5). The adverse health effects of air pollution on children include obesity, blood pressure (6), fetal and childhood growth retardation respiratory disorders (including asthma), developmental disorders, and an increased likelihood of developing cancer (7, 8). Some previous studies on the relationship between air pollution and childhood obesity had controversial findings.

Although imbalance between energy intake and consumption was the main cause of obesity in children and adolescents, also genetic, biological, and socio-environmental factors, such as dietary intake, physical activity (PA), and parental obesity, play key roles in the development of obesity in children and adolescents (2, 9–13). Since the air pollution incident in 2013, there were increasing research pieces of evidence that air pollution is associated with childhood obesity in recent years (7, 14–17). Among the four air pollutants compositions (NO_2_, O_3_, PM10, and PM2.5), previous studies found that PM2.5 had the greatest impact on obesity (14). Air pollutants mainly came from PM2.5 in Sichuan and Chongqing of China (18). Our current study found that the annual average PM2.5 concentration in Chongqing was 65.97 μg/m^3^, which was far exceeded the WHO air quality standard (WHO = 10 μg/m^3^).

Previous conclusions on the relationship between air pollution and childhood obesity were controversial. Some studies suggested that long-term exposure to PM2.5, NO_2_, and O_3_ was associated with obesity in children and adolescents (7, 14–17), and one study found no association between exposure to pollutants and childhood obesity (19), while the other study suggested that air pollution had a negative impact on childhood obesity (20). Since air pollution has a cumulative effect, there are no prior studies to confirm its dose-response and the growth curve of obesity under different PM2.5 exposure duration from pregnancy to adolescents. Furthermore, there are limited pieces of evidence from cohort studies (21). Therefore, this study intend to illustrate the hypothesis that long-term PM2.5 exposure is associated with the development of childhood obesity with a dose-response relationship.

This study will investigate the relationship between long-term PM2.5 exposure and childhood obesity based on two follow-ups and long-term PM2.5 exposure data from prenatal to 10–13 years old from a cohort of urban-rural areas in Chongqing. The impact of PM2.5 on five obesity indicators (body mass index (BMI), BMI Z-scores (BMIz), waist-to-height ratio (WHtR), overweight/obesity, and central obesity) and the impact of PM2.5 exposure during pregnancy on birth weight were also explored. Moreover, the growth curve of the obesity indicators under different PM2.5 exposure and the subgroup analyses of sex were also illustrated.

## Methods

### Participants and Visits

All subjects were recruited from Chongqing Children's Health Cohort, which was conducted at baseline in 2014–2015 and followed up in 2019. This study was approved by the Institutional Review Board of Children's Hospital of Chongqing Medical University (no. 2019-86).

This is an ongoing prospective cohort study to evaluate the dose relationship between PM2.5 and obesity. At baseline, a stratified cluster sampling of two stages was applied to select participants from two counties in Chongqing. One county represented the urban and the other represented the rural areas (stratification one) of Chongqing, respectively. Then, two avenues (stratification two) in each county were selected randomly, children of the resident families in these streets and studied in the primary schools were all included in this study (3, 6, 13, 22).

We recruited the participants who met the following criteria: (1) age range is 6–8 years at the baseline visit, (2) live in the target areas for more than 6 months, (3) without severe diseases (for instance, kidney disease, cardiovascular disease, or cancer), and all interviewees in this study were assented by both children and their parents to participate, and (4) providing a detailed home address. Questionnaires were used to recruit participants in public elementary schools. Finally, a total of 4,284 participants aged 6–8 years were included in this study (as shown in Supplementary Figure S1). At the baseline visit, we conducted the physical examination and questionnaire survey of the participants. Physical examination included height, weight, and waist circumference was organized by the elementary schools where the children and adolescents were studied. Nurses and doctors from the Children's Hospital of Chongqing Medical University participated in the physical examination, they were trained in rigorous accordance with the operation manual and passed the test before participating in the physical examination. Informed consent forms and questionnaires were distributed by the Children's Hospital of Chongqing Medical University. The questionnaire was completed by the parents or guardians of the children after standard training by the research group, and detailed instructions regarding the questionnaire were given to the parents or guardians. The questionnaires were collected by teachers of each class after completion. Demographic variables were included in the questionnaire, such as diet, PA, socioeconomic status (SES), birth weight, and disease history (3, 6, 13, 22).

At the followed up visit, physical examination was the same as at baseline, with the questionnaire adding environmental pollution and puberty development. The physical examination and questionnaire at the follow-up visit were conducted in the same way as at baseline.

### Physical Examination

Anthropometric measurements were carried out by pediatric nurses who experienced long-term training of more than 5 years. Height and weight were measured by trained nurses using a mobile ultrasound body machine (model: WS-H300D). Subjects were required to dress lightly, remove shoes and socks, and stand upright. The measurement environment was required to be quiet and spacious, relatively isolated, and free from bystanders, on a smooth and firm ground (3). For obesity indicators, BMI, which was calculated as weight (kg) divided by height squared (m^2^), was used as a criterion of general obesity. BMI_Z_ was calculated using the standard of Center for Disease Control (CDC) growth charts (23). A diagnosis of overweight or obesity was based on the sex-specific CDC BMI-for-age growth charts: overweight was defined as a BMI (BMI = weight [in kilograms]/height [in meters]^2^) at or above the 85th percentile and below the 95th percentile, and obesity was defined as a BMI at or above the 95th percentile (24). Additionally, an SAS code for the 2,000 CDC Growth Charts was used to diagnose overweight and obesity (25). Central obesity in children was assessed using the waist-height ratio (waist/height). Waist circumference was measured for children with fasting, standing upright with their feet together, and their abdomen relaxed. First, remove or lift their clothes and find their waist. Using fingers to find the top of the hip and the bottom of the rib cage. The lumbar area was the soft, fleshy part between these two bony parts. It was the narrowest part of the torso and was usually located at or above the belly button. Then, wrapped the measuring tape around the waist. Let children stand up straight and breathe normally and hold the end of the tape measure at the navel and turned it around the back to the front of the waist. The tape measure was paralleled to the floor and pressed against the torso without digging into the skin. Moreover, we ensured that the tape measure was straight all the way around and not twisted anywhere, especially in the back. Finally, we checked the measurements on the tape measure when participants exhaled. The waist measurement was where the zero end on the tape measure meet the slack end of the tape measure. Repeat the measurement one more time to ensure the accuracy of the original measurement. If it was different from the first time, a third time measure would be made and the average value of the three measures was used (3). If WHtR was ≥0.481 in male or ≥0.456 in female, they were diagnosed as central obesity, using the diagnostic criteria for Chinese children (26).

### Evaluation of the Accumulating Exposure to PM2.5

Each participant used a satellite-based PM2.5 concentration with a spatial resolution of 1 km. A machine-learning approach was used to estimate the monthly average PM2.5 concentrations for urban and rural areas of Chongqing from 2004 to 2019. The modeling method has been described in previous publications (27, 28). Moreover, for aerosol optical depth (AOD), the product provided by the National Aeronautics and Space Administration (NASA) was applied for multi-angle implementation of the atmospheric correction, which was described in previous studies (27, 29), to enhance the spatial resolution of PM2.5 estimates to 1 km. In order to assess the personal exposure level of PM2.5, the residential addresses collected from each participant during the baseline and follow-up visits were geocoded throughout the study period. According to the grid unit they live in, the monthly average PM2.5 concentration was assigned to participants at risk of increased weight during the follow-up. For participants who changed residential addresses during the tracking period, the monthly average concentration of PM2.5 at the addresses before and after the transfer of dwellings was used. The annual average concentration of PM2.5 was calculated following the steps described in the posterior. Firstly, the annual average concentration of PM2.5 was calculated as the weighted arithmetic mean of monthly average values of the current year. Secondly, the annual average of individual exposures to PM2.5 from pregnancy to the age at baseline visit and to the age at the follow-up visit was used. At last, the annual average exposure levels of PM2.5 from pregnancy to birth and to each year of age until the age of 10 were used as individual exposure level (6) (Table 1).

**Table 1:** The characteristics of participants at baseline.

**Variables**	**Total**	**Male**	**Female**	***P***
**Anthropometric variables**				
Age, years	7.87 ± 1.56	7.91 ± 1.56	7.83 ± 1.55	0.112
Waist, cm	56.00 ± 7.98	57.20 ± 8.55	54.72 ± 7.11	<0.001
Height, cm	127.42 ± 11.16	127.94 ± 11.00	126.87 ± 11.30	0.002
Weight, kg	27.57 ± 8.64	28.36 ± 8.96	26.74 ± 8.20	<0.001
BMI, kg/m^2^	16.65 ± 2.79	16.98 ± 2.78	16.31 ± 2.77	<0.001
BMI Z-score	0.15 ± 1.09	0.29 ± 1.14	0.00 ± 1.01	<0.001
WHtR	0.44 ± 0.05	0.45 ± 0.05	0.43 ± 0.05	<0.001
Birth weight, g	3, 281.80 ± 484.00	3, 339.80 ± 506.00	3, 224.00 ± 454.20	<0.001
Pregnancy week, weeks	39.15 ± 1.37	39.16 ± 1.40	39.13 ± 1.35	0.574
**PM2.5 level**, **μg/m**^**3**^				
In pregnancy	66.32 ± 7.28	66.23 ± 7.32	66.41 ± 7.24	0.434
~1 year old	66.68 ± 5.64	66.66 ± 5.65	66.70 ± 5.62	0.795
~2 year old	67.59 ± 5.80	67.55 ± 5.80	67.64 ± 5.80	0.576
~3 year old	68.28 ± 5.86	68.25 ± 5.88	68.30 ± 5.83	0.758
~4 year old	68.18 ± 5.67	68.20 ± 5.71	68.16 ± 5.62	0.816
~5 year old	67.69 ± 5.46	67.73 ± 5.51	67.65 ± 5.40	0.658
~6 year old	67.46 ± 5.37	67.49 ± 5.41	67.44 ± 5.33	0.780
~7 year old	67.28 ± 5.34	67.31 ± 5.38	67.26 ± 5.29	0.763
~8 year old	66.60 ± 5.34	66.65 ± 5.40	66.55 ± 5.28	0.569
~9 year old	65.60 ± 5.27	65.65 ± 5.35	65.54 ± 5.19	0.504
~10 year old	64.50 ± 5.12	64.55 ± 5.19	64.43 ± 5.04	0.439
Birth-weight^*^, g				
>4000g	1,468 (92.85)	748 (94.33)	720 (91.37)	0.023
≤ 4000g	113 (7.15)	45 (5.67)	68 (8.63)	
**Dietary intake, mean (g/day)**			
Cereals	181.05 ± 119.50	187.28 ± 123.10	174.36 ± 115.30	<0.001
Vegetables	196.37 ± 138.10	196.91 ± 138.70	195.80 ± 137.50	0.726
Red meat	151.51 ± 145.80	156.53 ± 147.10	146.12 ± 144.20	0.002
**Puberty**, ***n*** **(%)**				
No	3,168 (76.32)	1,158 (57.10)	2,010 (94.68)	<0.001
Yes	983 (23.68)	870 (42.90)	113 (5.32)	
**Physical activity**, ***n*** **(%)**				
0–3 days/week	1,759 (56.76)	868 (57.33)	891 (56.21)	0.530
4–7 days/week	1,340 (43.24)	646 (42.67)	694 (43.79)	
**Mother with obesity**, ***n*** **(%)**				
No	2, 788 (90.26)	1,348 (89.45)	1, 40 (91.02)	0.140
Yes	301 (9.74)	159 (10.55)	142 (8.98)	
**Passive smoking**, ***n*** **(%)**				
No	2,323 (60.06)	1,133 (59.54)	1,190 (60.56)	0.516
Yes	1,545 (39.94)	770 (40.46)	775 (39.44)	
**Household income**, ***n*** **(%)**				
~500	377 (9.26)	183 (9.21)	194 (9.31)	0.331
~1, 000	532 (13.07)	277 (13.93)	255 (12.24)	
~2, 000	851 (20.90)	411 (20.67)	440 (21.12)	
~3, 000	822 (20.19)	382 (19.22)	440 (21.12)	
>3, 000	1,489 (36.58)	743 (36.98)	754 (36.21)	

*BMI, body mass index; WHtR, weight to height ratio; PM2.5, an aerodynamic diameter of 2.5 μm or less*;

* *for the normal gestational age children*.

### Demographic Variables

Demographic information and SES were collected at baseline and follow-up. The validity and reliability of the demographic questionnaire were checked and detailed described in our published papers (3, 30, 31).

A history of disease for maternal obesity was also investigated using a self-filled questionnaire (self-reported height and weight). A quantitative food frequency questionnaire was used to collect dietary information, which has been described in detail in a previous publication (32). SES, such as household income, was investigated at baseline and follow-up visits. First, information on whether children were exposed to maternal active and passive smoking (PS) during pregnancy and the number of cigarettes smoked per day were surveyed. Second, at baseline and follow-up visits, the children's PS status was collected by the parental self-reported number of days smoked during the past 30 days and the number of cigarettes smoked per day (6). Individuals exposed to at least 5 parental cigarettes during pregnancy or at any visit in the past 30 days were considered to be in PS status. PA was assessed by your self-reported number of days per week doing enough PA to 'sweat' on or off-schools (33). In addition, PA information was collected under the cooperation of parents, teachers, and children to ensure the reliability of PA information, and the mean times of PA at baseline and follow-up visits were used to reflect the subjects′ PA level. The potential influence of puberty development was included. As puberty was not directly assessed (i.e., Tanner stages) in our study, we used spermarche/menarche (yes or no) as a surrogate measure (6). Information on spermarche/menarche was collected by questionnaire both from children and their parents to ensure the accuracy of it. Besides, pediatricians asked about the time and status of development of participants during physical examinations in schools at the follow-up in 2019.

### Statistical Analyses

Continuous variables are presented as mean ± SD, whereas categorical variables are presented as cases (n) and percentages (%). Anthropometric measurements and SES indexes between the male and female were assessed using Student's *t*-test and Chi-squared test. A mixed linear regression model was used to examine the association between long-term exposure to PM2.5 and obesity indexes (including BMI, BMI_Z_, and WHtR), which considered the repeated measurement of the multi-level data frame (33). The development of BMI, BMIz, and WHtR was explored by the use of individual growth curve modeling within a multilevel framework, which aimed to explore longitudinal data (33). Moreover, the increased absolute value of obesity indicators with each increase of 5 μg/m^3^ of PM2.5 was analyzed using a mixed linear regression model. In addition, a mixed logistic regression model was applied to study the dose-response relationship between PM2.5 exposure and the incidence of overweight/obesity and central obesity in two visits. The method to fit the mixed logistic regression model was as follows steps. First, model one adjusted age and sex. Second, covariates (i.e., puberty, maternal obesity, PS, PA, birth weight, and household income) were added to model one, from which model two was constituted. In addition, the subgroup of sex was analyzed. The odds ratio (OR) and 95% CIs of PM2.5 to obesity/central obesity were calculated based on every 5 μg/m^3^ increase in PM2.5 concentration.

The data analysis in this study was performed using SAS 9.4 software (Copyright © 2016 by SAS Institute Inc., Cary, NC, USA). We used the program SAS9.4 mixed model (proc mixed and proc glimmix) for multi-level modeling. The significant difference was defined as an α level of 0.05, with a two-sided test.

## Results

### General Characteristics

The demographic characteristics of the subjects at baseline are shown in Table 1, a total of 4,284 children were included. The average age at baseline was 7.87 ± 1.56 years, and 51.68% (2,214/4,284) were men. There were significant differences in BMI, BMIz, weight, waist circumference, and WHtR between male and female (all *p* < 0.05). In addition, the cumulative exposure levels of PM2.5 from pregnancy to 10 years old were also presented, and anthropometric indicators are shown in Table 1.

### The Risk of PM2.5 (per 5 μg/m^3^ Increase) Exposure on the Growth Curves of Obesity Indexes and Obesity

The PM2.5 exposure (per 5 μg/m^3^ increase) from pregnancy to age 6 and to two visits (2014–2015 at visit one and 2019 at visit two) on the growth curve of obesity indexes (i.e., BMI, BMIz, and WHtR) are analyzed by mixed linear regression model in Table 2. The results showed that a positive effect was found between the exposure of PM2.5 from pregnancy to 6 years old on the growth curves of WHtR [β (95% CIs): 0.007 (0.006, 0.009)], BMI [β (95% CIs): 0.128 (0.039, 0.217)], and BMIz [β (95% CIs): 0.073 (0.040, 0.106)] after adjusted covariates of age, sex, puberty, the mother with obesity, PS, PA, household income, dietary intake of cereals, vegetables, and red meat. In addition, the effects on BMI and BMIz were observed only in male by subgroup analyses of sex. While, the effect of PM2.5 exposure from pregnancy to two visits on the growth curve of obesity indexed was only significant on WHtR [β (95% CIs): 0.003 (0.002, 0.005)] after adjusted multi-covariates.

**Table 2:** The PM2.5 exposure (per 5 μg/m^3^) on the growth curve of obesity indexes.

**Variables**	**Total**		Male^*^		Female^*^	
	**β (95%CIs)**	***P***	**β (95%CIs)**	***P***	**β (95%CIs)**	***P***
**PM2.5 exposure to 6 years old**						
**WHtR**						
Model 1	0.009 (0.007, 0.010)	<0.001	0.008 (0.007, 0.010)	<0.001	0.008 (0.007, 0.010)	<0.001
Model 2	0.007 (0.006, 0.009)	<0.001	0.007 (0.005, 0.009)	<0.001	0.006 (0.004, 0.008)	<0.001
**BMI**						
Model 1	0.185 (0.114, 0.256)	<0.001	0.239 (0.138, 0.340)	<0.001	0.120 (0.027, 0.214)	0.012
Model 2	0.128 (0.039, 0.217)	0.005	0.216 (0.086, 0.346)	0.001	0.018 (-0.098, 0.133)	0.766
**BMIz**						
Model 1	0.084 (0.059, 0.110)	<0.001	0.102 (0.065, 0.140)	<0.001	0.057 (0.023, 0.091)	0.001
Model 2	0.073 (0.040, 0.106)	<0.001	0.100 (0.050, 0.149)	<0.001	0.032 (−0.010, 0.075)	0.134
**PM2.5 exposure to two visits**						
**WHtR**						
Model 1	0.005 (0.004, 0.006)	<0.001	0.003 (0.002, 0.005)	<0.001	0.006 (0.004, 0.007)	<0.001
Model 2	0.003 (0.002, 0.005)	<0.001	0.002 (0.000, 0.004)	0.027	0.004 (0.003, 0.006)	<0.001
**BMI**						
Model 1	0.039 (−0.023, 0.101)	0.217	−0.021 (−0.108, 0.066)	0.640	0.071 (−0.013, 0.155)	0.096
Model 2	−0.012 (−0.086, 0.062)	0.744	−0.049 (−0.158, 0.059)	0.370	0.001 (−0.096, 0.098)	0.985
**BMIz**						
Model 1	0.018 (−0.004, 0.040)	0.112	−0.010 (−0.041, 0.022)	0.554	0.026 (−0.002, 0.054)	0.069
Model 2	0.003 (−0.024, 0.030)	0.845	−0.017 (−0.057, 0.023)	0.414	0.006 (−0.028, 0.040)	0.725

*PM2.5, an aerodynamic diameter of 2.5 μm or less; WHtR, weight to height ratio; BMI, body mass index; BMIz, BMI Z score; two visits, the annual average of individual exposures to PM2.5 from pregnancy to the age at baseline visit and to the age at the follow-up visit. Model 1: adjusted age and sex. Model 2: adjusted age, sex, puberty, the mother with obesity, passive smoking, physical activity, household income, intake of cereals, vegetables, and red meat*.

* *adjusted age, puberty, the mother with obesity, passive smoking, physical activity, household income, dietary intake of cereals, vegetables, and red meat*.

The PM2.5 exposure (per 5 μg/m^3^ increase) on overweight/obesity and central obesity are analyzed using a mixed logistic regression model in Table 3. The effect of PM2.5 exposure from pregnancy to 6 years old was significant on the incidence of central obesity [OR (95% CIs): 1.26 (1.16, 1.37)], and the effect on overweight/obesity was not significant after adjusted covariates (*p* = 0.353). An increased risk of PM2.5 exposure from pregnancy to two visits on central obesity [OR (95% CIs): 1.21 (1.12, 1.31)] was observed, but the risk on overweight/obesity [OR (95% CIs):1.01 (0.94, 1.10), *p* = 0.729] was not significant.

**Table 3:** The effect of PM2.5 exposure (per 5 μg/m3) on overweight/obesity and central obesity.

**Model**	**Total**		Male^*^		Female^*^	
	**OR (95%CIs)**	***P***	**OR (95%CIs)**	***P***	**OR (95%CIs)**	***P***
**PM2.5 exposure from pregnancy to 6 years old**				
**Central obesity**				
Model 1	1.32 (1.24, 1.40)	<0.001	1.28 (1.17, 1.39)	<0.001	1.36 (1.25, 1.49)	<0.001
Model 2	1.26 (1.16, 1.37)	<0.001	1.26 (1.12, 1.42)	0.001	1.23 (1.09, 1.40)	0.001
**Overweight/obesity**				
Model 1	1.09 (1.03, 1.16)	0.006	1.09 (1.01, 1.18)	0.033	1.07 (0.97, 1.19)	0.150
Model 2	1.04 (0.96, 1.13)	0.353	1.10 (0.99, 1.23)	0.084	0.90 (0.78, 1.03)	0.111
**PM2.5 exposure from pregnancy to two visits**				
**Central obesity**				
Model 1	1.28 (1.21, 1.36)	<0.001	1.24 (1.14, 1.35)	<0.001	1.32 (1.22, 1.44)	<0.001
Model 2	1.21 (1.12, 1.31)	<0.001	1.16 (1.03, 1.30)	0.012	1.24 (1.11, 1.39)	<0.001
**Overweight/obesity**				
Model 1	1.07 (1.01, 1.14)	0.017	1.06 (0.98, 1.15)	0.168	1.04 (0.95, 1.14)	0.405
Model 2	1.01 (0.94, 1.10)	0.729	1.02 (0.91, 1.13)	0.759	0.91 (0.81, 1.03)	0.138

*PM2.5, an aerodynamic diameter of 2.5 μm or less; two visits: the annual average of individual exposures to PM2.5 from pregnancy to the age at baseline visit and to the age at the follow-up visit. Model 1: Adjusted age, sex. Model 2: Adjusted age, sex, puberty, the mother with obesity, passive smoking, physical activity, birth weight, household income, dietary intake of cereals, vegetables, and red meat*.

* *Adjusted age, puberty, the mother with obesity, passive smoking, physical activity, birth weight, household income, dietary intake of cereals, vegetables, and red meat*.

Compared PM2.5 exposure <60 μg/m^3^, children and adolescents living in PM2.5 exposure level for per 5 μg/m^3^ increase (PM2.5 exposure from pregnancy to 6 years old) had increased WHtR [β (95% CIs): 0.015 (0.011, 0.019)] after adjusting covariates, but the effect was not found in BMI and BMIz. The effect on WHtR was significant both in male [β (95% CIs): 0.013(0.006, 0.020)] and female [β (95% CIs): 0.016 (0.011, 0.022)] in the sex subgroup analyses (in Supplementary Table S1). Compared PM2.5 exposure <60 μg/m^3^, children and adolescents living in PM2.5 exposure level of 70–75 μg/m^3^ (PM2.5 exposure from pregnancy to two visits) had increased WHtR [β (95% CIs): 0.019 (0.014, 0.024)], BMI [β (95% CIs): 0.326 (0.037, 0.616)], and BMIz [β (95% CIs): 0.182 (0.074, 0.290)], and the effect on BMI and BMIz was only significant in male in the sex subgroup analyses (in Supplementary Table S1).

### The Dose-Response Relationship Between PM2.5 Exposure Duration and Obesity Indexes

The annual average exposure levels of PM2.5 from pregnancy to birth or to each year of age were used for individual exposure levels. The obesity indexes measured at the baseline visit (2014–2015) and follow-up visit (in 2019) were used to indicate which cumulative exposure period had the most significant impact on obesity indexes. The results showed that the cumulative effects of all age groups of PM2.5 exposure on BMI, BMIz, and WHtR were significant (all *p* < 0.05), and the effects were increased until participants were 6 years old (in Table 4), even after adjusting covariates. In subgroup analyses of sex, the cumulative effects of PM2.5 exposure on BMI and BMIz were significant in male (all *p* < 0.05). Moreover, the cumulative effects on WHtR were statistically significant in both male and female (all *p* < 0.001; in Table 4).

**Table 4:** Association between different PM2.5 exposure duration and BMI/BMI_Z_/WHtR in children and adolescents.

**Variables**	**PM2.5 level, μg/m^**3**^**	**Total**		**Male**		**Female**	
		**β (95%CIs)**	***P***	**β (95%CIs)**	***P***	**β (95%CIs)**	***P***
**Birthweight**					
	In pregnancy^*^	0.006 (−0.001, 0.013)	0.080	0.011 (0.001, 0.022)	0.032	0.001 (−0.009, 0.011)	0.840
	In pregnancy^#^	0.009 (0.001, 0.017)	0.037	0.012 (−0.000, 0.024)	0.052	0.005 (−0.006, 0.016)	0.352
**BMI**						
**Model 1**					
	In pregnancy	0.023 (0.012, 0.033)	<0.001	0.024 (0.009, 0.040)	0.002	0.021 (0.007, 0.036)	0.004
	~1 year old	0.036 (0.022, 0.050)	<0.001	0.039 (0.018, 0.059)	<0.001	0.031 (0.013, 0.050)	0.001
	~2 year old	0.039 (0.025, 0.052)	<0.001	0.043 (0.023, 0.063)	<0.001	0.033 (0.014, 0.051)	<0.001
	~3 year old	0.037 (0.023, 0.050)	<0.001	0.041 (0.021, 0.060)	<0.001	0.032 (0.014, 0.050)	<0.001
	~4 year old	0.036 (0.022, 0.051)	<0.001	0.040 (0.020, 0.060)	<0.001	0.031 (0.013, 0.050)	0.001
	~5 year old	0.037 (0.023, 0.052)	<0.001	0.040 (0.019, 0.062)	<0.001	0.032 (0.013, 0.052)	0.001
	**~** **6 year old**	**0.040 (0.025, 0.054)**	**<0.001**	**0.043 (0.022, 0.065)**	**<0.001**	**0.034 (0.014, 0.054)**	**<0.001**
	~7 year old	0.039 (0.024, 0.054)	<0.001	0.042 (0.021, 0.064)	<0.001	0.033 (0.013, 0.053)	0.001
	~8 year old	0.036 (0.021, 0.051)	<0.001	0.039 (0.018, 0.061)	<0.001	0.031 (0.011, 0.051)	0.002
	~9 year old	0.036 (0.020, 0.051)	<0.001	0.039 (0.017, 0.061)	<0.001	0.030 (0.010, 0.051)	0.003
	~10 year old	0.035 (0.019, 0.051)	<0.001	0.039 (0.017, 0.061)	<0.001	0.030 (0.009, 0.050)	0.005
**Model 2**					
	In pregnancy	0.012 (−0.002, 0.025)	0.089	0.015 (−0.005, 0.036)	0.135	0.006 (−0.012, 0.023)	0.531
	~1 year old	0.021 (0.003, 0.039)	0.021	0.030 (0.004, 0.057)	0.026	0.009 (−0.014, 0.032)	0.438
	~2 year old	0.027 (0.010, 0.045)	0.003	0.040 (0.013, 0.066)	0.003	0.012 (−0.011, 0.035)	0.299
	~3 year old	0.025 (0.007, 0.042)	0.006	0.036 (0.010, 0.062)	0.007	0.010 (−0.013, 0.032)	0.390
	~4 year old	0.023 (0.005, 0.041)	0.011	0.033 (0.007, 0.060)	0.015	0.008 (−0.015, 0.031)	0.497
	~5 year old	0.024 (0.005, 0.042)	0.014	0.034 (0.006, 0.061)	0.016	0.008 (−0.016, 0.032)	0.522
	**~** **6 year old**	**0.027 (0.008, 0.046)**	**0.006**	**0.038 (0.010, 0.066)**	**0.008**	**0.010 (−0.014, 0.035)**	**0.413**
	~7 year old	0.025 (0.006, 0.044)	0.010	0.036 (0.008, 0.064)	0.013	0.009 (−0.016, 0.034)	0.468
	~8 year old	0.022 (0.003, 0.041)	0.022	0.031 (0.003, 0.059)	0.028	0.007 (−0.018, 0.031)	0.605
	~9 year old	0.021 (0.002, 0.040)	0.033	0.031 (0.002, 0.059)	0.033	0.005 (−0.020, 0.030)	0.699
	~10 year old	0.020 (0.000, 0.040)	0.046	0.030 (0.001, 0.058)	0.044	0.004 (−0.022, 0.030)	0.765
**BMIz**						
**Model 1**					
	In pregnancy	0.010 (0.006, 0.014)	<0.001	0.011 (0.005, 0.017)	<0.001	0.009 (0.003, 0.014)	0.001
	~1 year old	0.016 (0.011, 0.021)	<0.001	0.017 (0.010, 0.025)	<0.001	0.013 (0.007, 0.020)	0.001
	~2 year old	0.017 (0.012, 0.022)	<0.001	0.018 (0.011, 0.026)	<0.001	0.014 (0.008, 0.021)	<0.001
	~3 year old	0.017 (0.012, 0.022)	<0.001	0.018 (0.011, 0.025)	<0.001	0.014 (0.008, 0.021)	<0.001
	~4 year old	0.017 (0.012, 0.022)	<0.001	0.018 (0.010, 0.025)	<0.001	0.014 (0.008, 0.021)	<0.001
	~5 year old	0.017 (0.012, 0.023)	<0.001	0.018 (0.011, 0.026)	<0.001	0.015 (0.008, 0.022)	<0.001
	**~** **6 year old**	**0.018 (0.013, 0.024)**	**<0.001**	**0.019 (0.011, 0.027)**	**<0.001**	**0.016 (0.008, 0.023)**	**<0.001**
	~7 year old	0.018 (0.012, 0.023)	<0.001	0.019 (0.011, 0.027)	<0.001	0.015 (0.008, 0.022)	<0.001
	~8 year old	0.017 (0.012, 0.023)	<0.001	0.018 (0.010, 0.026)	<0.001	0.015 (0.007, 0.022)	<0.001
	~9 year old	0.017 (0.011, 0.023)	<0.001	0.018 (0.010, 0.026)	<0.001	0.014 (0.007, 0.022)	<0.001
	~10 year old	0.017 (0.011, 0.023)	<0.001	0.018 (0.010, 0.026)	<0.001	0.014 (0.006, 0.022)	<0.001
**Model 2**					
	In pregnancy	0.008 (0.003, 0.013)	0.003	0.009 (0.001, 0.017)	0.027	0.005 (−0.001, 0.012)	0.104
	~1 year old	0.013 (0.006, 0.020)	<0.001	0.016 (0.005, 0.026)	0.003	0.009 (0.000, 0.017)	0.047
	~2 year old	0.016 (0.009, 0.022)	<0.001	0.019 (0.009, 0.029)	<0.001	0.010 (0.002, 0.019)	0.014
	~3 year old	0.015 (0.008, 0.021)	<0.001	0.018 (0.008, 0.028)	<0.001	0.010 (0.001, 0.018)	0.023
	~4 year old	0.014 (0.008, 0.021)	<0.001	0.017 (0.007, 0.027)	0.001	0.009 (0.000, 0.017)	0.047
	~5 year old	0.015 (0.008, 0.022)	<0.001	0.018 (0.007, 0.028)	0.001	0.009 (0.000, 0.018)	0.050
	**~** **6 year old**	**0.016 (0.009, 0.023)**	**<0.001**	**0.019 (0.008, 0.030)**	**<0.001**	**0.010 (0.001, 0.019)**	**0.029**
	~7 year old	0.015 (0.008, 0.022)	<0.001	0.018 (0.007, 0.029)	0.001	0.009 (0.000, 0.019)	0.043
	~8 year old	0.014 (0.007, 0.021)	<0.001	0.016 (0.006, 0.027)	0.003	0.008 (−0.001, 0.017)	0.081
	~9 year old	0.014 (0.006, 0.021)	<0.001	0.016 (0.005, 0.027)	0.004	0.008 (−0.002, 0.017)	0.107
	~10 year old	0.013 (0.006, 0.021)	<0.001	0.016 (0.005, 0.027)	0.005	0.007 (−0.002, 0.017)	0.136
**WHtR**		
**Model 1**		
	In pregnancy	0.001 (0.001, 0.001)	<0.001	0.001 (0.001, 0.001)	<0.001	0.001 (0.001, 0.001)	<0.001
	~1 year old	0.002 (0.001, 0.002)	<0.001	0.001 (0.001, 0.002)	<0.001	0.002 (0.001, 0.002)	<0.001
	~2 year old	0.002 (0.001, 0.002)	<0.001	0.002 (0.001, 0.002)	<0.001	0.002 (0.001, 0.002)	<0.001
	~3 year old	0.002 (0.001, 0.002)	<0.001	0.002 (0.001, 0.002)	<0.001	0.002 (0.001, 0.002)	<0.001
	~4 year old	0.002 (0.001, 0.002)	<0.001	0.002 (0.001, 0.002)	<0.001	0.002 (0.001, 0.002)	<0.001
	~5 year old	0.002 (0.002, 0.002)	<0.001	0.002 (0.001, 0.002)	<0.001	0.002 (0.002, 0.002)	<0.001
	**~** **6 year old**	**0.002 (0.002, 0.002)**	**<0.001**	**0.002 (0.001, 0.002)**	**<0.001**	**0.002 (0.002, 0.002)**	**<0.001**
	~7 year old	0.002 (0.002, 0.002)	<0.001	0.002 (0.001, 0.002)	<0.001	0.002 (0.002, 0.002)	<0.001
	~8 year old	0.002 (0.002, 0.002)	<0.001	0.002 (0.001, 0.002)	<0.001	0.002 (0.002, 0.002)	<0.001
	~9 year old	0.002 (0.002, 0.002)	<0.001	0.002 (0.001, 0.002)	<0.001	0.002 (0.002, 0.002)	<0.001
	~10 year old	0.002 (0.002, 0.002)	<0.001	0.002 (0.001, 0.002)	<0.001	0.002 (0.002, 0.002)	<0.001
**Model 2**					
	In pregnancy	0.001 (0.000, 0.001)	<0.001	0.001 (0.000, 0.001)	<0.001	0.001 (0.000, 0.001)	<0.001
	~1 year old	0.001 (0.001, 0.002)	<0.001	0.001 (0.001, 0.002)	<0.001	0.001 (0.001, 0.002)	<0.001
	~2 year old	0.001 (0.001, 0.002)	<0.001	0.001 (0.001, 0.002)	<0.001	0.001 (0.001, 0.002)	<0.001
	~3 year old	0.001 (0.001, 0.002)	<0.001	0.001 (0.001, 0.002)	<0.001	0.001 (0.001, 0.002)	<0.001
	~4 year old	0.001 (0.001, 0.002)	<0.001	0.001 (0.001, 0.002)	<0.001	0.001 (0.001, 0.002)	<0.001
	~5 year old	0.001 (0.001, 0.002)	<0.001	0.001 (0.001, 0.002)	<0.001	0.001 (0.001, 0.002)	<0.001
	**~** **6 year old**	**0.002 (0.001, 0.002)**	**<0.001**	**0.001 (0.001, 0.002)**	**<0.001**	**0.001 (0.001, 0.002)**	**<0.001**
	~7 year old	0.002 (0.001, 0.002)	<0.001	0.001 (0.001, 0.002)	<0.001	0.001 (0.001, 0.002)	<0.001
	~8 year old	0.001 (0.001, 0.002)	<0.001	0.001 (0.001, 0.002)	<0.001	0.001 (0.001, 0.002)	<0.001
	~9 year old	0.001 (0.001, 0.002)	<0.001	0.001 (0.001, 0.002)	<0.001	0.001 (0.001, 0.002)	<0.001
	~10 year old	0.001 (0.001, 0.002)	<0.001	0.001 (0.001, 0.002)	<0.001	0.001 (0.001, 0.002)	<0.001

*PM2.5, an aerodynamic diameter of 2.5 μm or less; WHtR, weight to height ratio; BMI, body mass index; BMIz, BMI Z score*;

* *adjusted gender*.

# *adjusted passive smoking, gender, the mother with obesity, PM2.5 level (In pregnancy), pregnancy week, pregnancy-induced hypertension. Model 1: adjusted age and sex. Model 2: adjusted age, sex (not included in sex subgroup analyses), puberty, the mother with obesity, passive smoking, physical activity, birth weight, household income, dietary intake of cereals, vegetables, and red meat. The bold values indicate the greatest cumulative effects of PM2.5 exposure on BMI, BMIz, and WHtR was at the age of six*.

Exposure to PM2.5 during pregnancy was positively correlated with the birth weight of normal gestational age children (β = 0.009, *p* = 0.037), after adjusting covariates. This association was more pronounced in boys (β = 0.012, *p* = 0.052) than in girls (β = 0.005, *p* = 0.352; Table 4).

### The Risk of PM2.5 Exposure Duration on the Incidence of Overweight/Obesity or Central Obesity

The mixed logistic regression model was used to analyze the risk of PM2.5 exposure on overweight/obesity or central obesity (Table 5). In model two, the effect of PM2.5 exposure on overweight/obesity was significant for participants with an exposure duration from 1 to 10 years old in the total sample [all *p* < 0.05, (except 1 and 10 years old; *p* = 0.057 and *p* = 0.064)], after adjusting covariates (Table 5), and the effect was only significant in male (*p* < 0.05). PM2.5 exposure duration from pregnancy to 10 years old was a significant risk factor for central obesity in the total sample (all *p* < 0.001). In addition, the effect was significant both in male [all *p* < 0.01 (except pregnancy in model 2, *p* = 0.052)] and in female (all *p* < 0.01; Table 5).

**Table 5:** The long-term effect of PM2.5 on overweight/obesity and central obesity using mixed logistic regression analyses.

**PM2.5 exposure**	**Total**		**Male**		**Female**	
	**OR (95%CIs)**	***P***	**OR (95%CIs)**	***P***	**OR (95%CIs)**	***P***
**Birthweight** **>** **4000g**					
In pregnancy^*^	1.01 (0.98, 1.04)	0.447	1.00 (0.97, 1.04)	0.867	1.02 (0.98, 1.07)	0.315
In pregnancy^#^	1.01 (0.98, 1.05)	0.447	1.02 (0.97, 1.06)	0.435	1.01 (0.95, 1.07)	0.745
**Overweight/obesity**					
**Model 1**						
In pregnancy	1.02 (1.01, 1.03)	<0.001	1.02 (1.01, 1.03)	0.004	1.02 (1.00, 1.03)	0.013
~1 year old	1.03 (1.02, 1.04)	<0.001	1.03 (1.01, 1.05)	<0.001	1.03 (1.01, 1.05)	0.008
~2 year old	1.03 (1.02, 1.04)	<0.001	1.03 (1.02, 1.05)	<0.001	1.03 (1.01, 1.05)	0.005
~3 year old	1.03 (1.02, 1.04)	<0.001	1.03 (1.01, 1.05)	<0.001	1.03 (1.01, 1.04)	0.007
~4 year old	1.03 (1.02, 1.04)	<0.001	1.03 (1.01, 1.05)	<0.001	1.03 (1.01, 1.05)	0.009
~5 year old	1.03 (1.02, 1.04)	<0.001	1.03 (1.01, 1.05)	<0.001	1.03 (1.01, 1.05)	0.012
~6 year old	1.03 (1.02, 1.04)	<0.001	1.03 (1.02, 1.05)	<0.001	1.03 (1.01, 1.05)	0.008
~7 year old	1.03 (1.02, 1.04)	<0.001	1.03 (1.02, 1.05)	<0.001	1.03 (1.01, 1.05)	0.010
~8 year old	1.03 (1.02, 1.04)	<0.001	1.03 (1.01, 1.05)	<0.001	1.03 (1.01, 1.05)	0.013
~9 year old	1.03 (1.02, 1.04)	<0.001	1.03 (1.01, 1.05)	<0.001	1.03 (1.00, 1.05)	0.018
~10 year old	1.03 (1.02, 1.04)	<0.001	1.03 (1.01, 1.05)	<0.001	1.02 (1.00, 1.05)	0.024
**Model 2**						
In pregnancy	1.01 (0.99, 1.02)	0.310	1.01 (1.00, 1.03)	0.152	1.00 (0.98, 1.02)	0.723
~1 year old	1.02 (1.00, 1.03)	0.057	1.03 (1.01, 1.05)	0.012	1.00 (0.97, 1.02)	0.735
~2 year old	1.02 (1.00, 1.04)	0.014	1.04 (1.01, 1.06)	0.002	1.00 (0.97, 1.02)	0.805
~3 year old	1.02 (1.00, 1.03)	0.032	1.03 (1.01, 1.05)	0.010	1.00 (0.98, 1.03)	0.913
~4 year old	1.02 (1.00, 1.03)	0.035	1.03 (1.01, 1.05)	0.014	1.00 (0.98, 1.03)	0.894
~5 year old	1.02 (1.00, 1.04)	0.033	1.03 (1.01, 1.06)	0.004	0.99 (0.97, 1.02)	0.539
~6 year old	1.02 (1.00, 1.04)	0.019	1.04 (1.01, 1.06)	0.002	0.99 (0.97, 1.02)	0.629
~7 year old	1.02 (1.00, 1.04)	0.021	1.03 (1.01, 1.05)	0.009	1.00 (0.98, 1.03)	0.771
~8 year old	1.02 (1.00, 1.04)	0.040	1.03 (1.01, 1.06)	0.005	0.99 (0.97, 1.02)	0.533
~9 year old	1.02 (1.00, 1.04)	0.041	1.03 (1.01, 1.06)	0.005	0.99 (0.96, 1.02)	0.483
~10 year old	1.02 (1.00, 1.04)	0.064	1.03 (1.01, 1.06)	0.008	0.99 (0.96, 1.02)	0.457
**Central obesity**					
**Model 1**					
In pregnancy	1.03 (1.02, 1.04)	<0.001	1.03 (1.01, 1.04)	<0.001	1.04 (1.03, 1.05)	<0.001
~1 year old	1.06 (1.04, 1.07)	<0.001	1.05 (1.03, 1.07)	<0.001	1.07 (1.05, 1.09)	<0.001
~2 year old	1.06 (1.05, 1.07)	<0.001	1.06 (1.04, 1.07)	<0.001	1.07 (1.05, 1.08)	<0.001
~3 year old	1.06 (1.05, 1.07)	<0.001	1.05 (1.04, 1.07)	<0.001	1.07 (1.05, 1.09)	<0.001
~4 year old	1.06 (1.05, 1.08)	<0.001	1.05 (1.04, 1.07)	<0.001	1.07 (1.05, 1.09)	<0.001
~5 year old	1.07 (1.05, 1.08)	<0.001	1.06 (1.04, 1.08)	<0.001	1.08 (1.06, 1.10)	<0.001
~6 year old	1.07 (1.05, 1.08)	<0.001	1.06 (1.04, 1.08)	<0.001	1.08 (1.06, 1.10)	<0.001
~7 year old	1.07 (1.05, 1.08)	<0.001	1.06 (1.04, 1.08)	<0.001	1.08 (1.06, 1.10)	<0.001
~8 year old	1.07 (1.05, 1.08)	<0.001	1.06 (1.04, 1.07)	<0.001	1.08 (1.06, 1.10)	<0.001
~9 year old	1.07 (1.05, 1.08)	<0.001	1.06 (1.04, 1.07)	<0.001	1.08 (1.06, 1.10)	<0.001
~10 year old	1.06 (1.05, 1.08)	<0.001	1.05 (1.04, 1.07)	<0.001	1.08 (1.06, 1.10)	<0.001
**Model 2**						
In pregnancy	1.02 (1.01, 1.03)	0.001	1.02 (1.00, 1.03)	0.052	1.02 (1.01, 1.04)	0.009
~1 year old	1.05 (1.03, 1.06)	<0.001	1.05 (1.03, 1.08)	<0.001	1.05 (1.02, 1.07)	<0.001
~2 year old	1.05 (1.03, 1.07)	<0.001	1.05 (1.03, 1.08)	<0.001	1.05 (1.03, 1.07)	<0.001
~3 year old	1.05 (1.03, 1.07)	<0.001	1.05 (1.03, 1.08)	<0.001	1.05 (1.03, 1.07)	<0.001
~4 year old	1.05 (1.03, 1.07)	<0.001	1.05 (1.03, 1.08)	<0.001	1.05 (1.03, 1.08)	<0.001
~5 year old	1.06 (1.04, 1.08)	<0.001	1.06 (1.03, 1.08)	<0.001	1.06 (1.03, 1.08)	<0.001
~6 year old	1.06 (1.04, 1.08)	<0.001	1.06 (1.03, 1.08)	<0.001	1.05 (1.03, 1.08)	<0.001
~7 year old	1.06 (1.04, 1.07)	<0.001	1.06 (1.03, 1.08)	<0.001	1.05 (1.03, 1.08)	<0.001
~8 year old	1.05 (1.03, 1.07)	<0.001	1.05 (1.03, 1.08)	<0.001	1.05 (1.03, 1.08)	<0.001
~9 year old	1.05 (1.04, 1.07)	<0.001	1.05 (1.03, 1.08)	<0.001	1.06 (1.03, 1.08)	<0.001
~10 year old	1.05 (1.03, 1.07)	<0.001	1.05 (1.02, 1.07)	0.0003	1.05 (1.02, 1.08)	0.0002

*PM2.5, an aerodynamic diameter of 2.5 μm or less*;

* *adjusted sex*.

# *adjusted sex, passive smoking, gestational weeks, maternal obesity, the father with obesity, gestational diabetes, gestational hypertension, mother's education. Model 1: adjusted age and sex. Model 2: adjusted age, sex (not included in sex subgroup analyses), puberty, the mother with obesity, passive smoking, physical activity, birth weight, household income, dietary intake of cereals, vegetables, and red meat*.

In addition, PM2.5 exposure during pregnancy was positively but not statistically significant [OR (95% CIs): 1.01 (0.98, 1.05), *p* = 0.447], associated with the incidence of newborns with birth weights over 4,000 g, after adjusting covariates (Table 5).

### The Effect of PM2.5 Exposure on the Growth Curve of the BMI and WHtR

**Figure 1:**
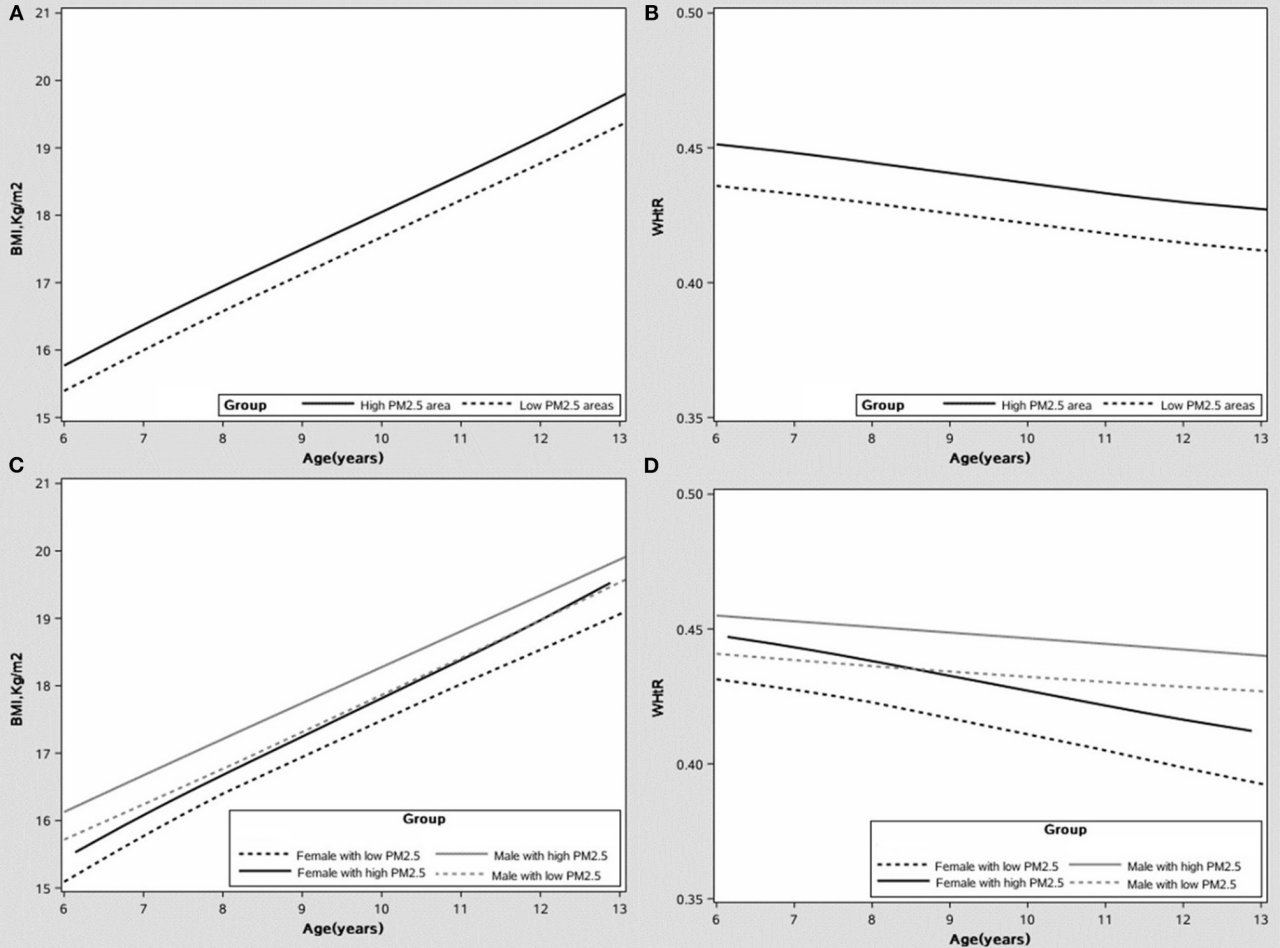
The growth curve of the body mass index (BMI) and waist-to-height ratio (WHtR) under different PM2.5 exposure levels, after adjusting covariates. **(A)** The growth curve of BMI. **(B)** The growth curve of WHtR. **(C)** The growth curve of BMI by sex. **(D)** The growth curve of WHtR by sex.

**Figure 2:**
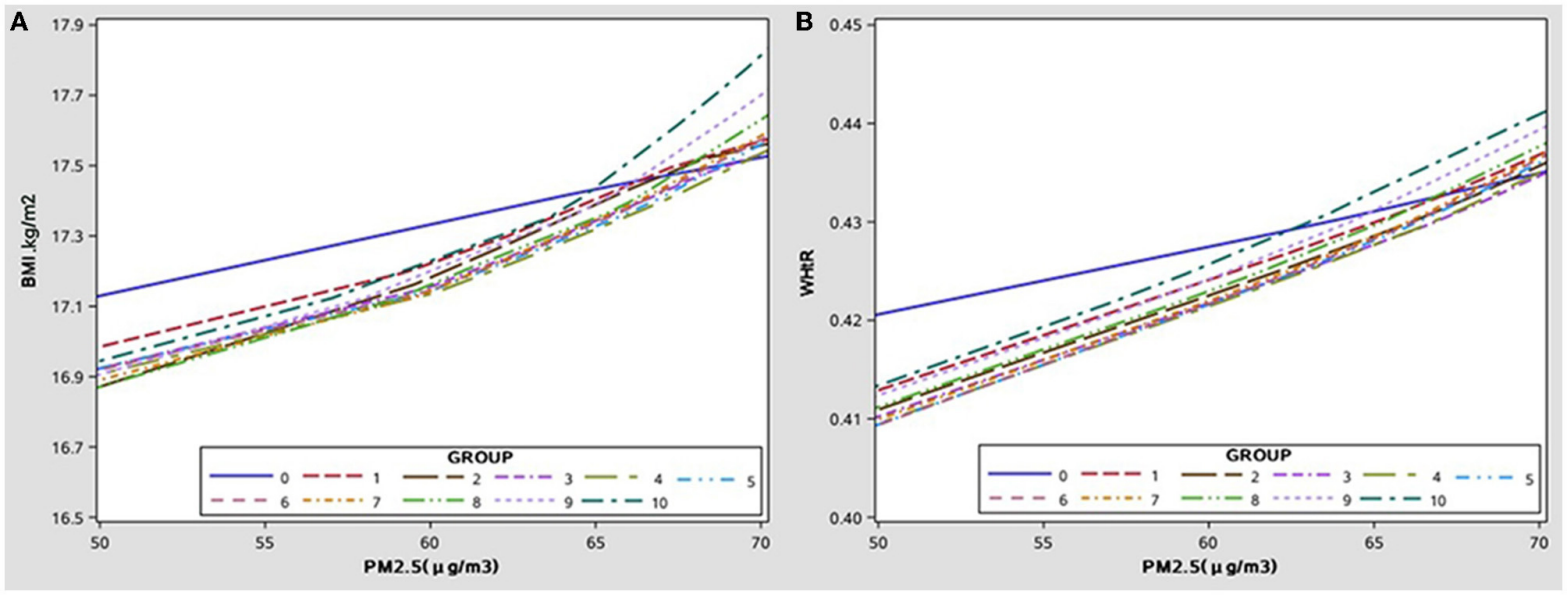
The cumulative effect of PM2.5 exposure levels to different ages on BMI/WHtR, after adjusting covariate. **(A)** The cumulative effect of PM2.5 on BMI with age. **(B)** The cumulative effect of PM2.5 on WHtR with age.

The growth curves of BMI and WHtR at high-low exposure levels of PM2.5 are shown in Figures 1A,B, respectively, and the subgroup analyses of growth curves by sex are shown in Figures 1C,D. From age 6 to 13, children exposed to high PM2.5 levels had higher BMI and WHtR when compared to those exposed to low PM2.5 levels (all *p* < 0.001) and this association was more significant in boys (in Figure 1; *p* < 0.001). The cumulative effect of PM2.5 on BMI and WHtR at different exposure duration was increased with increasing PM2.5 concentration (in Figure 2; all *p* < 0.001).

## Discussion

This study confirmed that PM2.5 exposure was associated with increased growth curves of BMI, BMIz, WHtR, and with increased prevalence of overweight/obesity and central obesity. Our study found that long-term exposure to high levels of PM2.5 was associated with the development of obesity in children and adolescents with a dose-response relationship. This effect was more pronounced in boys than in girls except for WHtR and illustrated the greatest significant impact at the age of six.

In recent years, the air pollution status in China has illustrated an obvious improvement, with PM2.5, PM10, and SO_2_ concentrations dropping significantly, but PM levels are still high compared with the developed countries (18). Our study found that the annual average PM2.5 concentration in Chongqing was 65.97 μg/m^3^ for children who were exposed from pregnancy to the follow-up, which was much higher than the annual average PM2.5 exposure concentration of the population-weighted annual average PM2.5 exposure concentration in China of 52.7 μg/m^3^ in 2017 (5, 34). Higher PM2.5 levels were associated with child health, especially obesity (7, 8, 16, 17, 35).

Exposure to PM2.5 during pregnancy was associated with an increased birth weight of normal gestational age boys and was not significantly associated with the occurrence of macrosomia (birth weight above 4,000 g) in our study. Some studies had suggested a negative association between PM2.5 exposure during pregnancy and birth weight and an association with increased risk of macrosomia (21, 36–39), which was inconsistent with our findings. A prospective cohort study in China suggested that PM2.5 exposure during pregnancy was linked to an increased prevalence of macrosomia (21). Our study found that PM2.5 exposure during pregnancy was positively, but not statistically, associated with the incidence of macrosomia, as our study did not distinguish the seasons of birth, which may explain the difference. In the United States, there was a significant positive association between low birth weight and PM2.5 exposure (throughout pregnancy or during a specific trimester) in the Mid-Atlantic, Mid-Northeast, and Mid-Northwest, and a significant negative association was found in the Mountain sub-region (37). The average PM2.5 level in that study (37) was 12.5 μg/m^3^, which was much lower than the level of 65.97 μg/m^3^ in our study. That may also partly account for the difference in results. Meanwhile, the effect of PM2.5 exposure during pregnancy on birth weight may vary by race and geography. Previous studies also showed significant differences in estimates of the effect of PM2.5 exposure on neonatal weight in studies with different exposure assessment methods, study designs, and study settings. Meanwhile, the accurate assessment of risk was limited by exposure measurements, demographics, and seasonal differences (36, 38).

According to the growth curve of the BMI and WHtR, children who were exposed to high PM2.5 levels had higher BMI or WHtR when compared to those exposed to low PM2.5 levels and this association was more significant in boys. The cumulative effect of PM2.5 on BMI or WHtR at different ages was increased with increasing PM2.5 concentration. To our knowledge, this was the first study to examine PM2.5 and the growth curves of obesity indicators in children and to analyze subgroup effects by sex. This study combined the growth and developmental characteristics of children with the cumulative effects of air pollution exposure, and our results provided a firm basis for the prevention and control of obesity growth in children.

The effects of different pollutants on obesity differed by sex. Our study and previous studies have found that the association between PM2.5 and childhood obesity was more significant in boys compared to girls (14, 16). In this study, the incidence of obesity was higher in boys. Probably because boys participate in more outdoor activities and have a greater lung capacity, and they inhale more PM2.5 than girls (40, 41). It may also be due to differences in growth and developmental characteristics, and the exact mechanism leading to the sex difference will be confirmed in future studies (42). The cumulative effect of PM2.5 on obesity was increased with age and was strongest after they were exposed to until 6 years old. PM2.5 concentration began to decrease when participants were 8 years old. As a handling method of the PM2.5 event in 2013, the Chinese government released the Air Pollution Prevention and Control Action Plan (18), which also illustrated an improved impact on obesity. Although the implementation of the Action Plan measures has been effective in improving air quality. However, air pollution in China remains severe and far exceeded the air quality standards issued by WHO (4).

Among the obesity indexes, PM2.5 had the strongest association with WHtR and central obesity. Therefore, it also suggested that we should not only focus on the weight of children because most of the fat in children is deposited in the abdomen (43). Our findings are consistent with many previous studies. A nationally cross-sectional study from 30 Chinese provinces showed that the exposure to PM2.5 was significantly associated with childhood obesity among 41,439 school-age children aged 6–17 years, BMI and PM2.5 exposure showed a significant positive correlation (7). A study from Spain confirmed a positive association between air pollution and obesity in children, and the exposure environment of home or school of other pollutants except PM2.5 did not show any significant correlation with BMI-z, and a significant relationship was found between PM2.5 exposure at school and BMI-z among 2,660 children aged 7–10 years (17). Indirectly, it was confirmed that PM2.5 has a significant effect on obesity (14). A study in Mexican children, adolescents, and adults showed an almost 2-fold increase in the odds of obesity for every 10 μg/m^3^ increase of PM2.5 (15). Besides, a study of adults in the UK also confirmed the correlation between PM2.5 and BMI (44). Most of the previous studies were cross-sectional and did not confirm the causal relationship between PM2.5 and obesity. Because air pollution has a cumulative effect, this study is the first study to examine the dose-response relationship between PM2.5 exposure from pregnancy to 10 years old and obesity in children and adolescents. Compared to other studies, this study is based on prospective multiple weight measurements and is the first study to analyze the effect of PM2.5 on growth curves of obesity indexes. In addition, a study from Jiangsu province, China, confirmed the relationship between ambient air pollutant exposure (3-, 4-, and 5-year average concentrations) and obesity in children and adolescents (14), while the exposure period was shorter than our study. However, results from an Italian birth cohort found that there was no relationship between exposure to air pollutants and obesity (e.g., BMI, blood lipids, and abdominal obesity) (19). The differences in conclusions may be due to differences in race and sample size of the study populations and differences in the types and concentration of pollutants and study design.

Currently, several studies illustrated the mechanism of how PM2.5 induced obesity. A previous study found that long-term PM2.5 exposure in mice leads to obesity *via* TLR4/Ikbke (45), which increased the risk of obesity and metabolic syndrome, and inflammatory activation regulated by TLR2/4 and lung lipid oxidation lead to abnormal metabolic function and obesity (46). The specific molecular biological mechanism remains to be confirmed in future studies. Besides, the positive association between PM2.5 and childhood obesity may be explained by the fact that air pollution may reduce children's outside PA time, leading to an imbalance between energy intake and expenditure (47).

### Strengths

Compared to previous studies, our study has three strengths. First, based on annual average PM2.5 exposures from pregnancy to adolescents, this is the first study to explore the dose-response relationship of PM2.5 and obesity for a long-term exposure window. Second, five obesity indicators were included to explore the correlation with PM2.5 exposure separately and to identify which indexes had the strongest association with PM2.5 exposure. Third, it is the first study to examine the growth curves of childhood obesity indicators at different PM2.5 exposure levels and durations.

### Limitations

This study has two limitations. First, our study measured only one type of pollutant and did not consider the association between other particles and obesity. Second, we measured the average PM2.5 concentration at home addresses and did not explore PM2.5 concentrations according to home addresses and schools separately. However, PM2.5 levels are the same in the same school, so there is a limited effect among individuals from different schools.

## Conclusion

Our findings confirm a dose-response relationship between PM2.5 exposure and childhood obesity, and this association is more pronounced in boys. Our findings represent a link between environmental conditions and children's health and provide a theoretical basis for future research on the etiology, intervention, and treatment of childhood obesity through environmental protection.

## Data Availability Statement

The original contributions presented in the study are included in the article/Supplementary Material, further inquiries can be directed to the corresponding author.

## Ethics Statement

The studies involving human participants were reviewed and approved by the Institutional Review Board at the Children's Hospital of Chongqing Medical University. Written informed consent to participate in this study was provided by the participants' legal guardian/next of kin.

## Author Contributions

XL: conceptualization, methodology, and writing—review and editing. FLiu and FLia: investigation and supervision. YR, XT, and DH: validation and resources. JT and YR: writing—original draft and writing—review and editing. XA: writing—review and editing. All authors critically reviewed and approved the final paper.

## Funding

This work was supported by the Major Health Project of Chongqing Science and Technology Bureau (No. CSTC2021jscx-gksb-N0001), Research and Innovation Team (No. W0088), National Key Research and Development Project (No. 2017YFC0211705), Intelligent Medicine Project (No. ZHYX202109), the Basic Research Project of Key Laboratory of Ministry of Education of China in 2021 (No. GBRP-202106), Joint Medical Research Project of Chongqing Municipal Health Commission and Chongqing Science and Technology Bureau (No. 2020MSXM062), the Technology Foresight and Institutional Innovation Project of Chongqing Science and Technology Bureau (No. cstc2020jsyj-zzysbAX0016), the Natural Science Foundation of Youth Project (No. 81502826), the Education Commission of Chongqing Municipality (No. KJQN201900443), the China Postdoctoral Science Foundation (No. 2014M562289), and Chongqing Postdoctoral Research Funded Projects (No. Xm2014129). The funders had no role in the study design, the data collection and analysis, the decision to publish, or the preparation of the manuscript.

## Conflict of Interest

The authors declare that the research was conducted in the absence of any commercial or financial relationships that could be construed as a potential conflict of interest.

## Publisher's Note

All claims expressed in this article are solely those of the authors and do not necessarily represent those of their affiliated organizations, or those of the publisher, the editors and the reviewers. Any product that may be evaluated in this article, or claim that may be made by its manufacturer, is not guaranteed or endorsed by the publisher.
