# Supplementary material for: The Impact of PM2.5 on the Growth Curves of Children's Obesity Indexes: A Prospective Cohort Study
Source: Front Public Health. 2022 Mar 22;10:843622. doi: 10.3389/fpubh.2022.843622 (PMC8980359; doi:10.3389/fpubh.2022.843622)
Supplement: Supplementary file 1 [file Table_1.DOCX]

| **Supplementary Table 1. The difference of obesity indexes for PM2.5 exposure by 5 μg/m^3^ increase** | | | | | | | | | |
| --- | --- | --- | --- | --- | --- | --- | --- | --- | --- |
| **Variables** | **60-65 μg/m^3^ vs. <60 μg/m^3^** | | |  | **65-70 μg/m^3^ vs. <60 μg/m^3^** | |  | **70-75 μg/m^3^ vs. <60 μg/m^3^** | |
|  | **Difference (95%CIs)** | | **P** |  | **Difference (95%CIs)** | **P** |  | **Difference (95%CIs)** | **P** |
| **Total sample** | |  | | |  |  |  |  |  |
| **PM2.5 exposure to 6 years old** | | | | |  |  |  |  |  |
| **WHtR** | | | | |  |  |  |  |  |
| Model 1 | **0.009(0.006,0.013)** | **<0.001** | | | **0.016(0.013,0.020)** | **<0.001** |  | **0.019(0.015,0.022)** | **<0.001** |
| Model 2 | **0.008(0.004,0.012)** | **<0.001** | | | **0.013(0.009,0.017)** | **<0.001** |  | **0.015(0.011,0.019)** | **<0.001** |
| **BMI** |  |  | | |  |  |  |  |  |
| Model 1 | -0.001(-0.195,0.193) | 0.990 | | | 0.054(-0.134,0.241) | 0.573 |  | 0.182(-0.025,0.389) | 0.086 |
| Model 2 | -0.054(-0.270,0.163) | 0.627 | | | -0.118(-0.339,0.103) | 0.296 |  | 0.042(-0.205,0.289) | 0.738 |
| **BMIz** |  |  | | |  |  |  |  |  |
| Model 1 | -0.017(-0.085,0.051) | 0.618 | | | 0.004(-0.063,0.071) | 0.913 |  | 0.076(0.002,0.150) | 0.043 |
| Model 2 | -0.028(-0.107,0.050) | 0.477 | | | -0.037(-0.118,0.044) | 0.370 |  | 0.038(-0.053,0.129) | 0.411 |
| **PM2.5 exposure to two visits** | | | | |  |  |  |  |  |
| **WHtR** | | | | |  |  |  |  |  |
| Model 1 | -0.003(-0.008,0.002) | 0.296 | | | 0.003(-0.002,0.007) | 0.228 |  | **0.021(0.017,0.025)** | **<0.001** |
| Model 2 | -0.001(-0.007,0.004) | 0.651 | | | 0.001(-0.004,0.007) | 0.633 |  | **0.019(0.014,0.024)** | **<0.001** |
| **BMI** |  |  | | |  |  |  |  |  |
| Model 1 | -0.059(-0.352,0.235) | 0.696 | | | 0.180(-0.093,0.453) | 0.196 |  | **0.466(0.231,0.701)** | **0.0001** |
| Model 2 | -0.050(-0.383,0.283) | 0.768 | | | 0.120(-0.189,0.430) | 0.446 |  | **0.326(0.037,0.616)** | **0.027** |
| **BMIz** |  |  | | |  |  |  |  |  |
| Model 1 | -0.065(-0.172,0.043) | 0.239 | | | 0.034(-0.066,0.134) | 0.506 |  | **0.198(0.112,0.284)** | **<0.001** |
| Model 2 | -0.048(-0.173,0.076) | 0.445 | | | 0.018(-0.098,0.134) | 0.763 |  | **0.182(0.074,0.290)** | **0.001** |
| **Male** |  |  | | |  |  |  |  |  |
| **PM2.5 exposure to 6 years old** | | | | |  |  |  |  |  |
| **WHtR** | | | | |  |  |  |  |  |
| Model 1 | **0.007(0.002,0.013)** | **0.005** | | | **0.015(0.010,0.020)** | **<0.001** |  | **0.016(0.010,0.021)** | **<0.001** |
| Model 2 | **0.008(0.002,0.014)** | **0.012** | | | **0.012(0.006,0.018)** | **<0.001** |  | **0.013(0.006,0.020)** | **0.0002** |
| **BMI** |  |  | | |  |  |  |  |  |
| Model 1 | -0.176(-0.457,0.105) | 0.22 | | | -0.025(-0.300,0.250) | 0.858 |  | -0.006(-0.307,0.295) | 0.970 |
| Model 2 | -0.141(-0.468,0.187) | 0.400 | | | -0.148(-0.484,0.189) | 0.389 |  | -0.082(-0.455,0.290) | 0.666 |
| **BMIz** |  |  | | |  |  |  |  |  |
| Model 1 | -0.107(-0.209,-0.005) | 0.040 | | | -0.035(-0.136,0.065) | 0.493 |  | -0.025(-0.135,0.085) | 0.659 |
| Model 2 | -0.088(-0.208,0.032) | 0.151 | | | -0.071(-0.196,0.054) | 0.266 |  | -0.042(-0.180,0.097) | 0.553 |
| **PM2.5 exposure to two visits** | | | | |  |  |  |  |  |
| **WHtR** | | | | |  |  |  |  |  |
| Model 1 | 0.001(-0.006,0.008) | 0.795 | | | 0.005(-0.001,0.012) | 0.105 |  | **0.022(0.017,0.028)** | **<0.001** |
| Model 2 | 0.002(-0.006,0.010) | 0.592 | | | 0.005(-0.003,0.013) | 0.198 |  | **0.021(0.013,0.028)** | **<0.001** |
| **BMI** |  |  | | |  |  |  |  |  |
| Model 1 | 0.244(-0.167,0.656) | 0.245 | | | **0.555(0.173,0.938)** | **0.004** |  | **0.719(0.388,1.050)** | **<0.001** |
| Model 2 | 0.240(-0.235,0.714) | 0.322 | | | **0.531(0.090,0.971)** | **0.018** |  | **0.647(0.228,1.066)** | **0.003** |
| **BMIz** |  |  | | |  |  |  |  |  |
| Model 1 | 0.089(-0.063,0.242) | 0.252 | | | **0.159(0.017,0.301)** | **0.028** |  | **0.302(0.179,0.425)** | **<0.001** |
| Model 2 | 0.099(-0.082,0.280) | 0.282 | | | **0.152(-0.016,0.319)** | **0.077** |  | **0.304(0.144,0.464)** | **0.0002** |
| **Female** |  |  | | |  |  |  |  |  |
| **PM2.5 exposure to 6 years old** | | | | |  |  |  |  |  |
| **WHtR** | | | | |  |  |  |  |  |
| Model 1 | **0.011(0.007,0.015)** | **<0.001** | | | **0.018(0.014,0.022)** | **<0.001** |  | **0.021(0.016,0.025)** | **<0.001** |
| Model 2 | **0.008(0.003,0.013)** | **0.002** | | | **0.013(0.008,0.018)** | **<0.001** |  | **0.016(0.011,0.022)** | **<0.001** |
| **BMI** |  |  | | |  |  |  |  |  |
| Model 1 | 0.082(-0.186,0.350) | 0.551 | | | 0.038(-0.210,0.286) | 0.763 |  | 0.259(-0.015,0.533) | 0.064 |
| Model 2 | -0.020(-0.309,0.269) | 0.894 | | | -0.191(-0.476,0.095) | 0.191 |  | 0.063(-0.253,0.380) | 0.696 |
| **BMIz** |  |  | | |  |  |  |  |  |
| Model 1 | 0.010(-0.078,0.098) | 0.820 | | | -0.016(-0.101,0.068) | 0.705 |  | 0.092(-0.000,0.185) | 0.051 |
| Model 2 | -0.021(-0.121,0.080) | 0.688 | | | -0.062(-0.163,0.039) | 0.226 |  | 0.036(-0.076,0.148) | 0.527 |
| **PM2.5 exposure to two visits** | | | | |  |  |  |  |  |
| **WHtR** | | | | |  |  |  |  |  |
| Model 1 | -0.005(-0.011,0.001) | 0.105 | | | 0.000(-0.006,0.006) | 0.965 |  | **0.020(0.015,0.025)** | **<0.001** |
| Model 2 | -0.004(-0.011,0.003) | 0.269 | | | -0.001(-0.008,0.005) | 0.667 |  | **0.017(0.010,0.023)** | **<0.001** |
| **BMI** |  |  | | |  |  |  |  |  |
| Model 1 | -0.326(-0.707,0.056) | 0.095 | | | -0.174(-0.525,0.177) | 0.332 |  | 0.199(-0.110,0.507) | 0.206 |
| Model 2 | -0.308(-0.731,0.115) | 0.153 | | | -0.215(-0.605,0.175) | 0.280 |  | -0.043(-0.415,0.330) | 0.822 |
| **BMIz** |  |  | | |  |  |  |  |  |
| Model 1 | **-0.208(-0.345,-0.071)** | **0.003** | | | -0.081(-0.207,0.045) | 0.206 |  | 0.078(-0.033,0.188) | 0.171 |
| Model 2 | **-0.173(-0.327,-0.018)** | **0.028** | | | -0.079(-0.222,0.063) | 0.276 |  | 0.037(-0.099,0.174) | 0.592 |
| two visits: the annual average of individual exposures to PM2.5 from pregnancy to the age at baseline visit and to the age at the follow-up visit  Model 1: Adjusted age and sex  Model 2: Adjusted age, sex (not included in sex subgroup analyses), puberty, mother with obesity, passive smoking, physical activity, birth-weight, household income, dietary intake of cereals, vegetables and red meat. | | | | | | | | | |
