# Supplementary figures and images for: The Impact of PM2.5 on the Growth Curves of Children's Obesity Indexes: A Prospective Cohort Study
Source: Front Public Health. 2022 Mar 22;10:843622. doi: 10.3389/fpubh.2022.843622 (PMC8980359; doi:10.3389/fpubh.2022.843622)

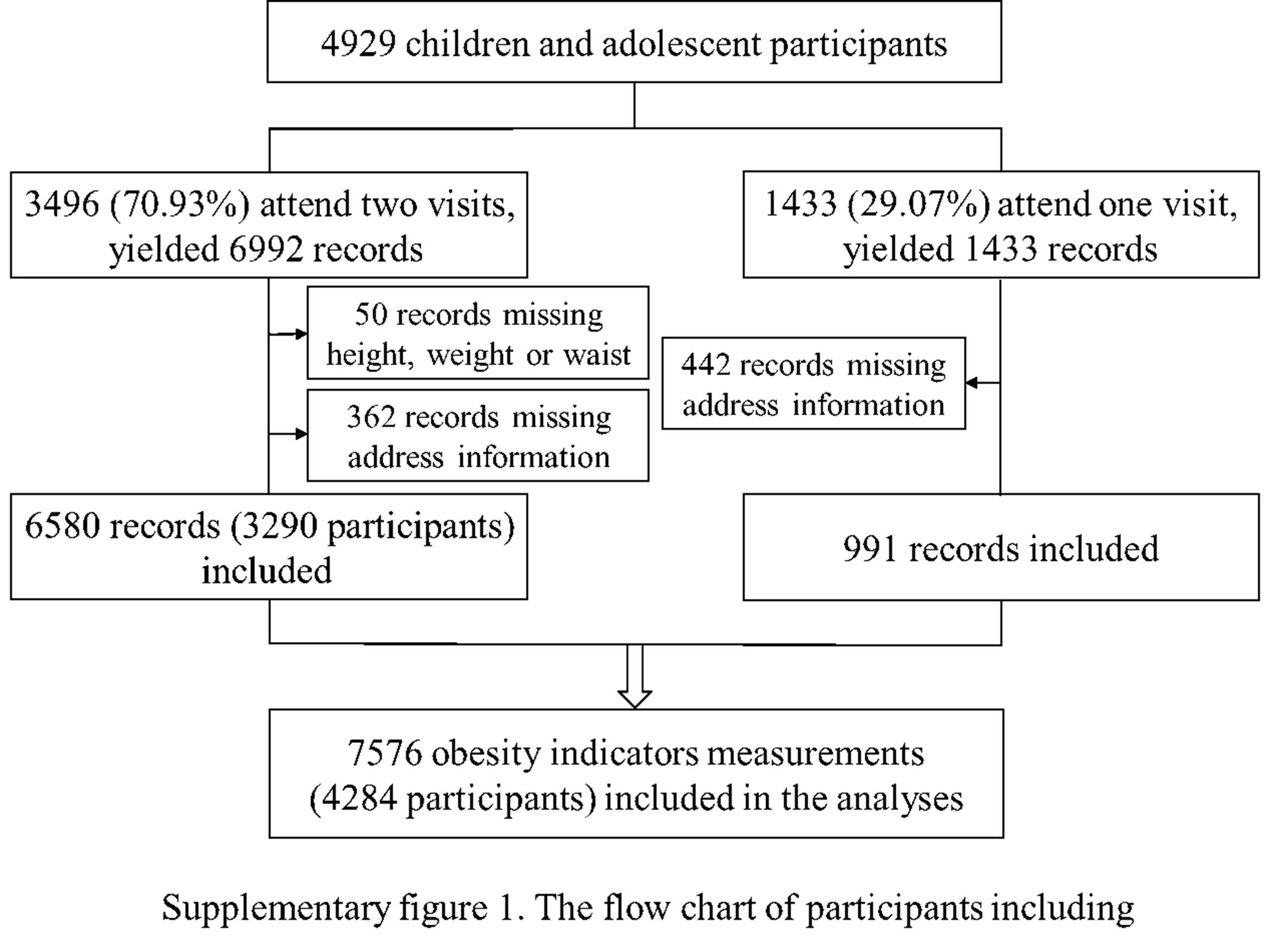

Supplement: Supplementary file 2 [file Image_1.PNG]
